# Supplementary material for: Precision prevention in worksite health–A scoping review on research trends and gaps
Source: PLoS One. 2024 Jun 10;19(6):e0304951. doi: 10.1371/journal.pone.0304951 (PMC11164362; doi:10.1371/journal.pone.0304951)
Supplement: S1 File — (PDF) [file pone.0304951.s001.pdf]

S1 Table 1. Search strings.

**Web of Science™**
(TI= (workplace or "work place" or worksite or "work site" or organisational or organizational or occupational* or worker or employee or corporate) AND TI=((precision or personali* or individuali* or stratif* or Tailo* or target*) NEAR/0(health or intervention or program or prevention or "health promotion"))) or (AB= (workplace or "work place" or worksite or "work site" or organisational or organizational or occupational* or worker or employee or corporate) AND AB=((precision or personali* or individuali* or stratif* or Tailo* or target*) NEAR/0(health or intervention or program or prevention or "health promotion")))
**Scopus®**
(TITLE (workplace OR "work place" OR worksite OR "work site" OR organisational OR organizational OR occupational* OR worker OR employee OR corporate) AND (TITLE (precision OR personali* OR individuali* OR stratif* OR tailo* OR target*) PRE/0 (health OR intervention OR program OR prevention OR "health promotion"))) OR (ABS (workplace OR "work place" OR worksite OR "work site" OR organisational OR organizational OR occupational* OR worker OR employee OR corporate) AND ABS ((precision OR personali* OR individuali* OR stratif* OR tailo* OR target*) PRE/0 (health OR intervention OR program OR prevention OR "health promotion")))

**Ovid MEDLINE®**	
**MEDLINE** health	(((workplace or work place or worksite or work site or organisational or organizational or occupational* or worker or employee or corporate) and ((precision or personali* or individuali* or stratif* or Tailo* or target*) adj health)).ti. or (workplace or work place or worksite or work site or organisational or organizational or occupational* or worker or employee or corporate).ab.) and

	((precision or personali* or individuali* or stratif* or Tailo* or target*) adj health).ab.
**MEDLINE** intervention	(((workplace or work place or worksite or work site or organisational or organizational or occupational* or worker or employee or corporate) and ((precision or personali* or individuali* or stratif* or Tailo* or target*) adj intervention)).ti. or (workplace or work place or worksite or work site or organisational or organizational or occupational* or worker or employee or corporate).ab.) and ((precision or personali* or individuali* or stratif* or Tailo* or target*) adj intervention).ab.
**MEDLINE** program	(((workplace or work place or worksite or work site or organisational or organizational or occupational* or worker or employee or corporate) and ((precision or personali* or individuali* or stratif* or Tailo* or target*) adj program)).ti. or (workplace or work place or worksite or work site or organisational or organizational or occupational* or worker or employee or corporate).ab.) and ((precision or personali* or individuali* or stratif* or Tailo* or target*) adj program).ab.
**MEDLINE** prevention	(((workplace or work place or worksite or work site or organisational or organizational or occupational* or worker or employee or corporate) and ((precision or personali* or individuali* or stratif* or Tailo* or target*) adj prevention)).ti. or (workplace or work place or worksite or work site or organisational or organizational or occupational* or worker or employee or corporate).ab.) and ((precision or personali* or individuali* or stratif* or Tailo* or target*) adj prevention).ab.

**MEDLINE** health promotion	(((workplace or work place or worksite or work site or organisational or organizational or occupational* or worker or employee or corporate) and ((precision or personali* or individuali* or stratif* or Tailo* or target*) adj "health promotion")).ti. or (workplace or work place or worksite or work site or organisational or organizational or occupational* or worker or employee or corporate).ab.) and ((precision or personali* or individuali* or stratif* or Tailo* or target*) adj "health promotion").ab.

**PubMed®**	
**PubMed** precision	(workplace[Title/Abstract] or "work place" [Title/Abstract] or worksite [Title/Abstract] or "work site" [Title/Abstract] organisational [Title/Abstract] or organizational [Title/Abstract] or occupational [Title/Abstract] or worker [Title/Abstract] or employee [Title/Abstract] or corporate [Title/Abstract]) and ("precision health" [Title/Abstract] or "precision prevention" [Title/Abstract] or "precision intervention" [Title/Abstract] or "precision program" [Title/Abstract] or "precision health promotion" [Title/Abstract])
**PubMed** personalized and personalised	(workplace[Title/Abstract] or "work place" [Title/Abstract] or worksite [Title/Abstract] or "work site" [Title/Abstract] organisational [Title/Abstract] or organizational [Title/Abstract] or occupational [Title/Abstract] or worker [Title/Abstract] or employee [Title/Abstract] or corporate [Title/Abstract]) and ("personalized health" [Title/Abstract] or "personalized prevention" [Title/Abstract] or "personalized intervention" [Title/Abstract] or "personalized program" [Title/Abstract] or "personalized health promotion" [Title/Abstract] or "personalised health" [Title/Abstract] or "personalised prevention"

	[Title/Abstract] or "personalised intervention" [Title/Abstract] or "personalised program" [Title/Abstract] or "personalised health promotion" [Title/Abstract])
**PubMed** individualized and individualised	(workplace[Title/Abstract] or "work place" [Title/Abstract] or worksite [Title/Abstract] or "work site" [Title/Abstract] organisational [Title/Abstract] or organizational [Title/Abstract] or occupational [Title/Abstract] or worker [Title/Abstract] or employee [Title/Abstract] or corporate [Title/Abstract]) and ("individualized health" [Title/Abstract] or " individualized prevention" [Title/Abstract] or " individualized intervention" [Title/Abstract] or " individualized program" [Title/Abstract] or " individualized health promotion" [Title/Abstract] or "individualised health" [Title/Abstract] or " individualised prevention" [Title/Abstract] or " individualised intervention" [Title/Abstract] or " individualised program" [Title/Abstract] or " individualised health promotion" [Title/Abstract])
**PubMed** stratified	(workplace [Title/Abstract] or "work place" [Title/Abstract] or worksite [Title/Abstract] or "work site" [Title/Abstract] organisational [Title/Abstract] or organizational [Title/Abstract] or occupational [Title/Abstract] or worker [Title/Abstract] or employee [Title/Abstract] or corporate [Title/Abstract]) and ("stratified health" [Title/Abstract] or " stratified prevention" [Title/Abstract] or " stratified intervention" [Title/Abstract] or " stratified program" [Title/Abstract] or " stratified health promotion" [Title/Abstract])
**PubMed** tailored	(workplace[Title/Abstract] or "work place" [Title/Abstract] or worksite [Title/Abstract] or "work site" [Title/Abstract] organisational [Title/Abstract] or organizational [Title/Abstract] or occupational [Title/Abstract] or worker [Title/Abstract] or employee [Title/Abstract] or corporate [Title/Abstract]) and ("tailored health" [Title/Abstract] or " tailored prevention" [Title/Abstract] or " tailored intervention" [Title/Abstract] or " tailored program" [Title/Abstract] or " tailored health promotion" [Title/Abstract])

**PubMed** targeted	(workplace[Title/Abstract] or "work place" [Title/Abstract] or worksite [Title/Abstract] or "work site" [Title/Abstract] organisational [Title/Abstract] or organizational [Title/Abstract] or occupational [Title/Abstract] or worker [Title/Abstract] or employee [Title/Abstract] or corporate [Title/Abstract]) and ("targeted health" [Title/Abstract] or " targeted prevention" [Title/Abstract] or " targeted intervention" [Title/Abstract] or " targeted program" [Title/Abstract] or " targeted health promotion" [Title/Abstract])

**APA PsychInfo® (via Scopus®)**	
personalized	AB (workplace or work place or worksite or work site or organisational or organizational or occupational* or worker or employee or corporate) AND AB (“personalized health” or “personalized intervention” or “personalized program” or “personalized prevention” or “personalized health promotion”) OR TI (workplace or work place or worksite or work site or organisational or organizational or occupational* or worker or employee or corporate) AND TI(“personalized health” or “personalized intervention” or “personalized program” or “personalized prevention” or “personalized health promotion”)
personalised	AB (workplace or work place or worksite or work site or organisational or organizational or occupational* or worker or employee or corporate) AND AB (“personalised health” or “personalised intervention” or “personalised program” or “personalised prevention” or “personalised health promotion”) OR TI (workplace or work place or worksite or work site or organisational or organizational or occupational* or worker or employee or corporate) AND TI(“personalised health” or “personalised intervention” or “personalised program” or “personalised prevention” or “personalised health promotion”)
precision	AB (workplace or work place or worksite or work site or organisational or organizational or occupational* or worker or employee or corporate) AND AB (

	“precision health” or “precision intervention” or “precision program” or “precision prevention” or “precision health promotion”) OR TI (workplace or work place or worksite or work site or organisational or organizational or occupational* or worker or employee or corporate) AND TI(“precision health” or “precision intervention” or “precision program” or “precision prevention” or “precision health promotion”)
targeted	AB (workplace or work place or worksite or work site or organisational or organizational or occupational* or worker or employee or corporate) AND AB (“targeted health” or “targeted intervention” or “targeted program” or “targeted prevention” or “targeted health promotion”) OR TI (workplace or work place or worksite or work site or organisational or organizational or occupational* or worker or employee or corporate) AND TI(“targeted health” or “targeted intervention” or “targeted program” or “targeted prevention” or “targeted health promotion”)
tailored	AB (workplace or work place or worksite or work site or organisational or organizational or occupational* or worker or employee or corporate) AND AB (“tailored health” or “tailored intervention” or “tailored program” or “tailored prevention” or “tailored health promotion”) OR TI (workplace or work place or worksite or work site or organisational or organizational or occupational* or worker or employee or corporate) AND TI(“tailored health” or “tailored intervention” or “tailored program” or “tailored prevention” or “tailored health promotion”)
stratified	AB (workplace or work place or worksite or work site or organisational or organizational or occupational* or worker or employee or corporate) AND AB (“stratified health” or “stratified intervention” or “stratified program” or “stratified prevention” or “stratified health promotion”) OR TI (workplace or work place or worksite or work site or organisational or organizational or occupational* or worker or employee or corporate) AND TI(“stratified health” or “stratified

	intervention” or “stratified program” or “stratified prevention” or “stratified health promotion”)
individualized	AB (workplace or work place or worksite or work site or organisational or organizational or occupational* or worker or employee or corporate) AND AB (“individualized health” or “individualized intervention” or “individualized program” or “individualized prevention” or “individualized health promotion”) OR TI (workplace or work place or worksite or work site or organisational or organizational or occupational* or worker or employee or corporate) AND TI(“individualized health” or “individualized intervention” or “individualized program” or “individualized prevention” or “individualized health promotion”)
individualised	AB (workplace or work place or worksite or work site or organisational or organizational or occupational* or worker or employee or corporate) AND AB (“individualised health” or “individualised intervention” or “individualised program” or “individualised prevention” or “individualised health promotion”) OR TI (workplace or work place or worksite or work site or organisational or organizational or occupational* or worker or employee or corporate) AND TI(“individualised health” or “individualised intervention” or “individualised program” or “individualised prevention” or “individualised health promotion”)

**Embase® (via OVID)**	
health	((workplace or work place or worksite or work site or organisational or organizational or occupational* or worker or employee or corporate) and ((precision or personali* or individuali* or stratif* or Tailo* or target*) adj health)).ti. or ((workplace or work place or worksite or work site or organisational or organizational or occupational* or worker or employee or

	corporate) and ((precision or personali* or individuali* or stratif* or Tailo* or target*) adj health)).ab.
intervention	((workplace or work place or worksite or work site or organisational or organizational or occupational* or worker or employee or corporate) and ((precision or personali* or individuali* or stratif* or Tailo* or target*) adj intervention)).ti. or ((workplace or work place or worksite or work site or organisational or organizational or occupational* or worker or employee or corporate) and ((precision or personali* or individuali* or stratif* or Tailo* or target*) adj intervention)).ab.
program	((workplace or work place or worksite or work site or organisational or organizational or occupational* or worker or employee or corporate) and ((precision or personali* or individuali* or stratif* or Tailo* or target*) adj program)).ti. or ((workplace or work place or worksite or work site or organisational or organizational or occupational* or worker or employee or corporate) and ((precision or personali* or individuali* or stratif* or Tailo* or target*) adj program)).ab.
prevention	((workplace or work place or worksite or work site or organisational or organizational or occupational* or worker or employee or corporate) and ((precision or personali* or individuali* or stratif* or Tailo* or target*) adj prevention)).ti. or ((workplace or work place or worksite or work site or organisational or organizational or occupational* or worker or employee or corporate) and ((precision or personali* or individuali* or stratif* or Tailo* or target*) adj prevention)).ab.
health promotion	((workplace or work place or worksite or work site or organisational or organizational or occupational* or worker or employee or corporate) and

	((precision or personali* or individuali* or stratif* or Tailo* or target*) adj health promotion)).ti. or ((workplace or work place or worksite or work site or organisational or organizational or occupational* or worker or employee or corporate) and ((precision or personali* or individuali* or stratif* or Tailo* or target*) adj health promotion)).ab.

S1. Table 2. Eligibility criteria.

	Inclusion	Exclusion
Population	- Human participants: workers, employee, trainee etc. in "working context" (workplace, worksite, organization, occupation etc.) of any organizations/companies etc.<br>- Analysis of historical datasets (e.g, health records, epidemiological datasets)<br>- Human samples (e.g., tissue samples, genetic material)	- Human participants outside the "working context" (e.g., community, school, health care settings: patients, military etc.); children<br>- Evaluation of new technologies that do not include human participants in the working context (cf. Ryan et al., 2021)<br>- human/sports performance outcomes (e.g., physical conditioning programs for healthy athletes)
Concept	- Studies that refer to the concept of precision prevention or its derivates (cf. search term)<br>- Any study collecting health-related clinical, psychosocial, behavioral, environmental, work-related information (e.g. weight loss, disease prevalence/risk, healthy diet, physical activity, sedentariness, mental health)	- Non-health outcomes including economic outcomes (e.g., cost-effectiveness studies),<br>- Animal studies
Context	- Any geographical location<br>- Setting: workplace etc. (see. above)	- "non-occupational settings / other settings" (community, school etc.)
Types of evidence	- Primary empirical research studies (e.g., RCTs, cohort studies, cross, sectional studies, and case reports)<br>- Protocols for planned studies<br>- Full-text articles available in electronic or hard-copy<br>- Full-text conference proceedings<br>- Articles written in English<br>- Peer-reviewed journal papers	- Reviews, meta-analyses (e.g., systematic, narrative, scoping reviews)<br>- Editorial articles (e.g., position statements)<br>- Protocols for reviews<br>- Abstracts or posters<br>- Articles: full-text not available in electronic or hard-copy<br>- Articles (full text) not written in English<br>- Dissertations, book chapters, books etc.
