# Supplementary material for: Precision prevention in worksite health–A scoping review on research trends and gaps
Source: PLoS One. 2024 Jun 10;19(6):e0304951. doi: 10.1371/journal.pone.0304951 (PMC11164362; doi:10.1371/journal.pone.0304951)
Supplement: S2 File — (PDF) [file pone.0304951.s002.pdf]

S2. Reference list of included articles.

1. Abbas SZ, Pollard TM, Wynn P, Learmonth A, Joyce K, Bambra C. The effectiveness of using the workplace to identify and address modifiable health risk factors in deprived populations. Occup Environ Med. 2015;72(9):664-9 DOI: 10.1136/oemed-2014-102743.

2. Ammar S, Daud A, Ismail AF, Razali A. Efficacy of a Targeted Intervention Method to Improve the Use of Hearing Protection Devices among Agro-Industrial Workers in Malaysia. Appl Sci. 2022;12(5) DOI: 10.3390/app12052497.

3. Andersen LN, Juul-Kristensen B, Roessler KK, Herborg LG, Sørensen TL, Søgaard K. Efficacy of 'Tailored Physical Activity' in reducing sickness absence among health care workers: design of a randomised controlled trial. BMC Public Health. 2013;13(1):917 DOI: 10.1186/1471-2458-13-917.

4. Andersen LN, Juul-Kristensen B, Roessler KK, Herborg LG, Sørensen TL, Søgaard K. Efficacy of ‘Tailored Physical Activity’ on reducing sickness absence among health care workers: A 3-months randomised controlled trial. Man Ther. 2015;20(5):666-71 DOI: 10.1016/j.math.2015.04.017.

5. Andersen LN, Juul-Kristensen B, Sørensen TL, Herborg LG, Roessler KK, Søgaard K. Efficacy of Tailored Physical Activity or Chronic Pain Self-Management Programme on return to work for sick-listed citizens: A 3-month randomised controlled trial. Scand J Public Health. 2015;43(7):694-703 DOI: 10.1177/1403494815591687.

6. Asfar T, Arheart KL, McClure LA, Ruano-Herreria EC, Dietz NA, Ward KD, et al. Implementing a Novel Workplace Smoking Cessation Intervention Targeting Hispanic/Latino Construction Workers: A Pilot Cluster Randomized Trial. Health Educ Behav. 2021;48(6):795-804 DOI: 10.1177/1090198120960395.

7. Asfar T, Caban-Martinez AJ, McClure LA, Ruano-Herreria EC, Sierra D, Clark GG, et al. A cluster randomized pilot trial of a tailored worksite smoking cessation intervention targeting Hispanic/Latino construction workers: Intervention development and research design. Contemp Clin Trials. 2018;67:47-55 DOI: 10.1016/j.cct.2018.02.007.

8. Baumann H, Heuel L, Bischoff LL, Wollesen B. Efficacy of Individualized Sensory-Based mHealth Interventions to Improve Distress Coping in Healthcare Professionals: A Multi-Arm Parallel-Group Randomized Controlled Trial. Sensors. 2023;23(4):2322 DOI: 10.3390/s23042322.

9. Beer-Borst S, Hayoz S, Eisenblatter J, Jent S, Siegenthaler S, Strazzullo P, et al. RE-AIM evaluation of a one-year trial of a combined educational and environmental workplace intervention to lower salt intake in Switzerland. Prev Med Rep. 2019;16 DOI: 10.1016/j.pmedr.2019.100982.

10. Berninger NM, Plasqui G, Crutzen R, Ruiter RAC, Kok G, Ten Hoor GA. The Effects of UPcomplish on Office Workers' Sedentary Behaviour, Quality of Life and Psychosocial Determinants: A Stepped-Wedge Design. Int J Behav Med. 2022;29(6):728-42 DOI: 10.1007/s12529-022-10054-0.

11. Berninger NM, Ten Hoor GA, Plasqui G, Kok G, Peters GY, Ruiter RAC. Sedentary Work in Desk-Dominated Environments: A Data-Driven Intervention Using Intervention Mapping. JMIR Form Res. 2020;4(7):e14951 DOI: 10.2196/14951.

12. Bidargaddi N, Almirall D, Murphy S, Nahum-Shani I, Kovalcik M, Pituch T, et al. To Prompt or Not to Prompt? A Microrandomized Trial of Time-Varying Push Notifications to Increase Proximal Engagement With a Mobile Health App. JMIR MHealth UHealth. 2018;6(11):e10123 DOI: 10.2196/10123.

13. Blake H, Bennett E, Batt ME. Evaluation of occupational health checks for hospital employees. Int J Workplace Health Manag. 2014;7(4):247-66 DOI: 10.1108/IJWHM-07-2013-0027.

14. Blake H, Hussain B, J, Rowl, s D, Juma A, et al. Employee perceptions of a workplace HIV testing intervention. Int J Workplace Health Manag. 2018;11(5):333-48 DOI: 10.1108/IJWHM-03-2018-0030.

15. Bonn S, Licitra G, Bellocco R, Lagerros YT. Clinical Outcomes Among Working Adults Using the Health Integrator Smartphone App: Analyses of Prespecified Secondary Outcomes in a Randomized Controlled Trial. J Med Internet Res. 2022;24(3) DOI: 10.2196/24725.

16. Bonn SE, Lof M, Ostenson CG, Lagerros YT. App-technology to improve lifestyle behaviors among working adults - the Health Integrator study, a randomized controlled trial. BMC Public Health. 2019;19 DOI: 10.1186/s12889-019-6595-6.

17. Broneder E, Wagner F, Weiß C, Fritz J, Sili M, Arendse M. mHealthINX –A virtual reality-based occupational stress management solution for older employees. HCI International 2021 - Posters, 23rd HCI International Conference, Virtual Event, 2021 Jul 24-29, Proceedings, Part I. 2021. p. 249-256 DOI: 10.1007/978-3-030-78635-9_34

18. Caperchione CM, Sharp P, Bottorff JL, Stolp S, Oliffe JL, Johnson ST, et al. The POWERPLAY workplace physical activity and nutrition intervention for men: Study protocol and baseline characteristics. Contemp Clin Trials. 2015;44:42-7 DOI: 10.1016/j.cct.2015.07.013.

19. Caperchione CM, Stolp S, Bottorff JL, Oliffe JL, Johnson ST, Seaton C, et al. Changes in Men's Physical Activity and Healthy Eating Knowledge and Behavior as a Result of Program Exposure: Findings From the Workplace POWERPLAY Program. J Phys Act Health. 2016;13(12):1364-71 DOI: 10.1123/jpah.2016-0111.

20. Chadyiwa M, Kagura J, Stewart A. Investigating Machine Learning Applications in the Prediction of Occupational Injuries in South African National Parks. Mach Learn Knowl. 2022;4(3):768-78 DOI: 10.3390/make4030037.

21. Choi SH, Waltje AH, Ronis DL, Noonan D, Hong O, Richardson CR, et al. Web-Enhanced Tobacco Tactics With Telephone Support Versus 1-800-QUIT-NOW Telephone Line Intervention for Operating Engineers: Randomized Controlled Trial. J Med Internet Res. 2014;16(11) DOI: 10.2196/jmir.3375.

22. Christensen JR, Faber A, Ekner D, Overgaard K, Holtermann A, Søgaard K. Diet, physical exercise and cognitive behavioral training as a combined workplace based intervention to reduce body weight and increase physical capacity in health care workers - a randomized controlled trial. BMC Public Health. 2011;11(1):671- DOI: 10.1186/1471-2458-11-671.

23. Christensen JR, Overgaard K, Carneiro IG, Holtermann A, Søgaard K. Weight loss among female health care workers- a 1-year workplace based randomized controlled trial in the FINALE-health study. BMC Public Health. 2012;12(1):625 DOI: 10.1186/1471-2458-12-625.

24. Damen I, Van Den Heuvel R, Brankaert R, Vos S, editors. Advancing Digital Behavior Change Interventions by Exploring a Calendar-Based Suggestion System. ECCE Proceedings of the 32nd European Conference on Cognitive Ergonomics; 2021 Apr 26-29; New York, USA. pp. 1-7. DOI: 10.1145/3452853.3452866.

25. Das BM, Petruzzello SJ. The use of active living every day to improve mass transit district employees' physical activity affect and enjoyment. Int J Health Promot Educ. 2014;53(3):147-55 DOI: 10.1080/14635240.2014.978349.

26. Dashti HS, Alimenti K, Levy DE, Hivert MF, McCurley JL, Saxena R, et al. Chronotype Polygenic Score and the Timing and Quality of Workplace Cafeteria Purchases: Secondary Analysis of the ChooseWell 365 Randomized Controlled Trial. Curr Dev Nutr. 2023;7(3) DOI: 10.1016/j.cdnut.2023.100048.

27. De Cocker K, Cardon G, Bennie JA, Kolbe-Alexander T, Meester F, Vandelanotte C. From Evidence-Based Research to Practice-Based Evidence: Disseminating a Web-Based Computer-Tailored Workplace Sitting Intervention through a Health Promotion Organisation. Int J Environ Res Public Health. 2018;15(5):1049-NA DOI: 10.3390/ijerph15051049.

28. De Cocker K, Cardon G, Vergeer I, Radtke T, Vandelanotte C. Who uses action planning in a web-based computer-tailored intervention to reduce workplace sitting and what do action plans look like? Analyses of the Start to stand intervention among Flemish employees. Appl Psychol Health Well-Being. 2019;11(3):543-61 DOI: 10.1111/aphw.12167.

29. De Cocker K, De Bourdeaudhuij I, Cardon G, Vandelanotte C. The Effectiveness of a Web-Based Computer-Tailored Intervention on Workplace Sitting: A Randomized Controlled Trial. J Med Internet Res. 2016;18(5):e96 DOI: 10.2196/jmir.5266.

30. De Cocker K, De Bourdeaudhuij I, Cardon G, Vandelanotte C. Theory-driven, web-based, computer-tailored advice to reduce and interrupt sitting at work: Development, feasibility and acceptability testing among employees. BMC Public Health. 2015;15(1) DOI: 10.1186/s12889-015-2288-y.

31. De Cocker K, De Bourdeaudhuij I, Cardon G, Vandelanotte C. What are the working mechanisms of a web-based workplace sitting intervention targeting psychosocial factors and action planning? BMC public health. 2017;17(1):382 DOI: 10.1186/s12889-017-4325-5.

32. Dias L, Eertmans A, Van den Brande I, Handaja Y, Taeymans S, et al. Using an Interactive Self-Assessment Tool to Strengthen Your Employee Assistance Service. J. Workplace Behav Health. 2015;30(1):46-65 DOI: 10.1080/15555240.2015.998974.

33. Doda D, Rothmore P, Pisaniello D, Briggs N, Stewart S, Mahmood M, et al. Relative benefit of a Stage of Change approach for the prevention of musculoskeletal pain and discomfort: A cluster randomised trial. Occup Environ Med. 2015;72(11):784-91 DOI: 10.1136/oemed-2015-102916.

34. Duan YP, Shang BR, Liang W, Yang M, Brehm W. Psychosocial profiles of physical activity fluctuation in office employees: A latent profile analysis. Plos One. 2020;15(1) DOI: 10.1371/journal.pone.0227182.

35. Elvitigala DS, Scholl PM, Suriyaarachchi H, Dissanayake V, Nanayakkara S, editors. StressShoe: A DIY Toolkit for just-in-time Personalised Stress Interventions for Office Workers Performing Sedentary Tasks. Proceedings of MobileHCI 2021 - The ACM International Conference on Mobile Human-Computer Interaction; 2021 Sept 27–Oct 1; Toulouse & Virtual France. New York, USA: Association for Comuting Machinery. DOI: proceedings/10.1145/3447526

36. Filon FL, MacUlan P, Crivellaro MA, Mauro M. Effectiveness of a Skin Care Program With a Cream Containing Ceramide C and a Personalized Training for Secondary Prevention of Hand Contact Dermatitis. Dermatitis. 2023;34(2):127-34 DOI: 10.1089/derm.2022.29002.flf.

37. Gans KM, Risica PM, Dulin-Keita A, Mello J, Dawood M, Strolla LO, et al. Innovative video tailoring for dietary change: Final results of the Good for you! cluster randomized trial. Int. J. Behav. Nutr. Phys. Act. 2015;12 DOI: 10.1186/s12966-015-0282-5.

38. Gellert GA, Montgomery S, Gellert TE, Gillett J, Kerekes A, Hole J. Employee Wellness in a Changing Climate: Environmental Heat Stress Driving Need for Targeted Health Promotion and Risk Reduction. J. Ecophysiol. Occup. Health. 2022;22(2):60-6 DOI: 10.18311/JEOH/2022/29945.

39. Gram B, Holtermann A, Søgaard K, Sjøgaard G. Effect of individualized worksite exercise training on aerobic capacity and muscle strength among construction workers--a randomized controlled intervention study. Scand J Work Environ Health. 2012;38(5):467-75 DOI: 10.5271/sjweh.3260.

40. Green S, Markaki A, Baird J, Murray P, Edwards R. Addressing Healthcare Professional Burnout: A Quality Improvement Intervention. Worldviews Evid. -Based Nurs. 2020;17(3):213-20 DOI: 10.1111/wvn.12450.

41. Grigorescu S, Cazan A-M, Rogozea L, Grigorescu DO. Original targeted therapy for the management of the burnout syndrome in nurses: an innovative approach and a new opportunity in the context of predictive, preventive and personalized medicine. EPMA J. 2020;11(2):161-76 DOI: 10.1007/s13167-020-00201-6.

42. Grigorescu S, Cazan AM, Grigorescu OD, Rogozea LM. The role of the personality traits and work characteristics in the prediction of the burnout syndrome among nurses-a new approach within predictive, preventive, and personalized medicine concept. EPMA J. 2018;9(4):355-65 DOI: 10.1007/s13167-018-0151-9.

43. Gryshchuk L, Campbell MA, Brunelle C, Doyle JN, Nero JW. Profiles of vulnerability to alcohol use and mental health concerns in first responders. J Police Crim Psychol. 2022;37(4):952-61 DOI: 10.1007/s11896-022-09546-2.

44. Haslam C, Kazi A, Duncan M, Clemes S, Twumasi R. Walking Works Wonders: A tailored workplace intervention evaluated over 24 months. Ergonomics. 2019;62(1):31-41 DOI: 10.1080/00140139.2018.1489982.

45. Haslam C, Kazi A, Duncan M, Twumasi R, Clemes S. Walking Works Wonders: A Workplace Health Intervention Evaluated Over 24 Months. Proceedings of the 20th Congress of the International Ergonomics Association; 2018 August 27-30; Florence, Italy. Cham: Springer International Publishing; 2019. pp. 1571-8. DOI: 10.1007/978-3-319-96071-5_161

46. Hees HL, de Vries G, Koeter MW, Schene AH. Adjuvant occupational therapy improves long-term depression recovery and return-to-work in good health in sick-listed employees with major depression: results of a randomised controlled trial. Occup Environ Med. 2013;70(4):252-60 DOI: 10.1136/oemed-2012-100789.

47. Hees HL, Koeter MWJ, de Vries G, Ooteman W, Schene AH. Effectiveness of adjuvant occupational therapy in employees with depression: design of a randomized controlled trial. BMC Public Health. 2010;10, 558 DOI: 10.1186/1471-2458-10-558.

48. Hnizdo E, Berry A, Hakobyan A, Beeckman-Wagner LA, Catlett L. Worksite wellness program for respiratory disease prevention in heavy-construction workers. J Occup Environ Med. 2011;53(3):274-81 DOI: 10.1097/JOM.0b013e31820b0ab1.

49. Holtermann A, Jorgensen MB, Gram B, Christensen JR, Faber A, Overgaard K, et al. Worksite interventions for preventing physical deterioration among employees in job-groups with high physical work demands: Background, design and conceptual model of FINALE. BMC Public Health. 2010;10 DOI: 10.1186/1471-2458-10-120.

50. Hong OS, Eakin BL, Chin DL, Feld J, Vogel S. An Internet-Based Tailored Hearing Protection Intervention for Firefighters: Development Process and Users' Feedback. Health Promot Pract. 2013;14(4):572-9 DOI: 10.1177/1524839912462031.

51. Huang JJ, Lin HS, Yen MF, Kan WM, Lee BO, Chen CH. Effects of a Workplace Multiple Cardiovascular Disease Risks Reduction Program. Asian Nurs Res. 2013;7(2):74-82 DOI: 10.1016/j.anr.2013.04.001.

52. Hutchinson J, Headley S, Matthews T, Spicer G, Dempsey K, Wooley S, et al. Changes in sitting time and sitting fragmentation after a workplace sedentary behaviour intervention. Int. J. Environ. Res. Public Health. 2018;15(6) DOI: 10.3390/ijerph15061148.

53. Jay K, Brandt M, Hansen K, Sundstrup E, Jakobsen MD, Schraefel MC, et al. Effect of Individually Tailored Biopsychosocial Workplace Interventions on Chronic Musculoskeletal Pain and Stress Among Laboratory Technicians: Randomized Controlled Trial. Pain Physician. 2015;18(5):459-71.

54. Jay K, Brandt M, Sundstrup E, Schraefel M, Jakobsen MD, Sjøgaard G, et al. Effect of individually tailored biopsychosocial workplace interventions on chronic musculoskeletal pain, stress and work ability among laboratory technicians: randomized controlled trial protocol. BMC Musculoskelet Disord. 2014;15:444 DOI: 10.1186/1471-2474-15-444.

55. Jeelani I, Albert A, Han K, Azevedo R. Are Visual Search Patterns Predictive of Hazard Recognition Performance? Empirical Investigation Using Eye-Tracking Technology. J Constr Eng Manag. 2018;145(1) DOI: 10.1061/(ASCE)CO.1943-7862.0001589.

56. Jensen JD, King AJ, Carcioppolo N, Krakow M, Samadder NJ, Morgan S. Comparing tailored and narrative worksite interventions at increasing colonoscopy adherence in adults 50-75: A randomized controlled trial. Soc Sci Med. 2014;104:31-40 DOI: 10.1016/j.socscimed.2013.12.003.

57. Jenull BB, Wiedermann W. The Different Facets of Work Stress: A Latent Profile Analysis of Nurses' Work Demands. J Appl Gerontol. 2015;34(7):823-43 DOI: 10.1177/0733464813495472.

58. Jones-Bitton A, Gillis D, Peterson M, McKee H. Latent burnout profiles of veterinarians in Canada: Findings from a cross-sectional study. Vet Rec. 2023;192(2): e2281 DOI: 10.1002/vetr.2281.

59. Kapanen AI, Conklin AI, Gobis B, Leung L, Yuen J, Zed PJ. Pharmacist-led cardiovascular risk prevention in Western Canada: a qualitative study. Int J Pharm Pract. 2021;29(1):45-54 DOI: 10.1111/ijpp.12658.

60. Kim JY, Oh S, Steinhubl S, Kim S, Bae WK, Han JS, et al. Effectiveness of 6 months of tailored text message reminders for obese male participants in a worksite weight loss program: randomized controlled trial. JMIR Mhealth Uhealth. 2015;3(1):e14 DOI: 10.2196/mhealth.3949.

61. Kocielnik R, Sidorova N. Personalized Stress Management: Enabling Stress Monitoring with LifelogExplorer. Kunstl Intell. 2015;29(2):115-22 DOI: 10.1007/s13218-015-0348-1.

62. Kouwenhoven-Pasmooij TA, Robroek SJW, Kraaijenhagen RA, Helmhout PH, Nieboer D, Burdorf A, et al. Effectiveness of the blended-care lifestyle intervention 'PerfectFit': a cluster randomised trial in employees at risk for cardiovascular diseases. BMC Public Health. 2018;18 DOI: 10.1186/s12889-018-5633-0.

63. Kraft D, Schmidt A, Büttner L, Oschinsky FM, Lambusch F, Van Laerhoven K, et al., editors. CareCam: Towards user-tailored Interventions at the Workplace using a Webcam. Paper presented at: PETRA '22: The15th International Conference on PErvasive Technologies Related to Assistive Environments. 29 June – 1 July; 2022 Corfu, Greece DOI: 10.1145/3529190.3534778.

64. Kwasnicka D, Vandelanotte C, Rebar A, Gardner B, Short C, Duncan M, et al. Comparing motivational, self-regulatory and habitual processes in a computer-tailored physical activity intervention in hospital employees - protocol for the PATHS randomised controlled trial. BMC Public Health. 2017;17(1):518 DOI: 10.1186/s12889-017-4415-4.

65. Kwasnicka D, Vandelanotte C, Rebar A, Gardner B, Short C, Duncan M, et al. Physical activity tailored intervention in hospital (PATHS): A Randomised controlled trial on online tailored program. Ann. Behav. Med. 2020; 53(S1):166.

66. Lamb JJ, Stone M, D'Adamo C R, Volkov A, Metti D, Aronica L, et al. Personalized Lifestyle Intervention and Functional Evaluation Health Outcomes SurvEy: Presentation of the LIFEHOUSE Study Using N-of-One Tent-Umbrella-Bucket Design. J. Pers. Med. 2022;12(1):115 DOI: 10.3390/jpm12010115.

67. Langille JL, Berry TR, Reade IL, Witcher C, Loitz CC, Rodgers WM. Strength of messaging in changing attitudes in a workplace wellness program. Health Promot Pract. 2011;12(2):303-11 DOI: 10.1177/1524839909336650. Epub 2009 Jun 16.

68. Lawanot W, Inoue M, Yokemura T, Mongkolnam P, Nukoolkit C, editors. Daily Stress and Mood Recognition System Using Deep Learning and Fuzzy Clustering for Promoting Better Well-Being. 2019. Proceedings of the 2019 IEEE International Conference on Consumer Electronics (ICCE); 2019 Jan 11 – 13; Vegas, NV, USA; pp. 1-6 DOI: 10.1109/ICCE.2019.8661932

69. Leduc C, Giga SI, Fletcher IJ, Young M, Dorman SC. Effectiveness of fitness training and psychosocial education intervention programs in wildland firefighting: a cluster randomised control trial. Int J Wildland Fire. 2022;31(8):799-815 DOI: 10.1071/WF21126.

70. Leduc C, Giga SI, Fletcher IJ, Young M, Dorman SC. Participatory Development Process of Two Human Dimension Intervention Programs to Foster Physical Fitness and Psychological Health and Well-Being in Wildland Firefighting. Int J Environ Res Public Health. 2021;18(13) DOI: 10.3390/ijerph18137118.

71. Lenz TL, Monaghan MS. Implementing lifestyle medicine with medication therapy management services to improve patient-centered health care. J Am Pharm Assoc (2003). 201;51(2):184-8 DOI: 10.1331/JAPhA.2011.10169.

72. Leung CLK, Li KK, Wei VWI, Tang A, Wong SYS, Lee SS, et al. Profiling vaccine believers and skeptics in nurses: A latent profile analysis. Int J Nurs Stud. 2022;126 DOI: 10.1016/j.ijnurstu.2021.104142. Epub 2021 Nov 27.

73. Levy DE, Gelsomin ED, Rimm EB, Pachucki M, Sanford J, Anderson E, et al. Design of ChooseWell 365: Randomized controlled trial of an automated, personalized worksite intervention to promote healthy food choices and prevent weight gain. Contemp Clin Trials. 2018;75:78-86 DOI: 10.1016/j.cct.2018.11.004. Epub 2018 Nov 7

74. Li J, Yu H, He S, Xue M, Tian D, Zhou J, et al. The association between awareness and behavior concerning the need for protection when using pesticide sprays and neurologic symptoms: A latent class cluster analysis. Medicine (Baltimore). 2019;98(30):e16588 DOI: 10.1097/MD.0000000000016588.

75. Lieslehto J, Rantanen N, Oksanen LMAH, Oksanen SA, Kivimäki A, Paju S, et al. A machine learning approach to predict resilience and sickness absence in the healthcare workforce during the COVID-19 pandemic. Sci Rep. 2022;12(1) DOI: 10.1038/s41598-022-12107-6.

76. Loeppke R, Edington D, Bender J, Reynolds A. The association of technology in a workplace wellness program with health risk factor reduction. J Occup Environ Med. 2013;55(3):259-64 DOI: 10.1097/JOM.0b013e3182898639.

77. Mauriello LM, Gkbayrak NS, Van Marter DF, Paiva AL, Prochaska JM. An internet-based computer-tailored intervention to promote responsible drinking: Findings from a pilot test with employed adults. Alcohol Treat Q. 2011;30(1):91-108 DOI: 10.1080/07347324.2012.635528.

78. McCall HC, Beahm JD, Fournier AK, Burnett JL, Carleton RN, Hadjistavropoulos HD. Stakeholder perspectives on internet-delivered cognitive behavioural therapy for public safety personnel: A qualitative analysis. Canadian Journal of Behavioural Science. 2021;53(3):232-42 DOI: 10.1037/cbs0000242.

79. McGuckin T, Sealey R, Barnett F, Walla P. The use and evaluation of a theory-informed, multi-component intervention to reduce sedentary behaviour in the workplace. Cogent Psychol. 2017;4(1) DOI: 10.1080/23311908.2017.1411038.

80. McHugh J, Suggs LS. Online tailored weight management in the worksite: Does it make a difference in biennial health risk assessment data? J Health Commun. 2012;17(3):278-93 DOI: 10.1080/10810730.2011.626496.

81. Mistretta EG, Davis MC, Temkit M, Lorenz C, Darby B, Stonnington CM. Resilience Training for Work-Related Stress Among Health Care Workers: Results of a Randomized Clinical Trial Comparing In-Person and Smartphone-Delivered Interventions. J Occup Environ Med. 2018;60(6):559-68 DOI: 10.1097/jom.0000000000001285.

82. Niks I, de Jonge J, Gevers J, Houtman I. Work Stress Interventions in Hospital Care: Effectiveness of the DISCovery Method. Int J Environ Res Public Health. 2018;15(2):332 DOI: 10.3390/ijerph15020332.

83. Nylen EC, Lindfors P, Ishall L, Goransson S, Aronsson G, Kylin C, et al. A pilot-study of a worksite based participatory intervention program: Its acceptability and short-term effects on work climate and attitudes in human service employees. Work. 2017;56(4):625-636 DOI: 10.3233/wor-172522.

84. Otto AK, Wollesen B. Multicomponent exercises to prevent and reduce back pain in elderly care nurses: a randomized controlled trial. BMC Sports Sci Med Rehabil. 2022;14(1) DOI: 10.1186/s13102-022-00508-z.

85. Ouellette RR, Frazier SL, Shernoff ES, Cappella E, Mehta TG, Maríñez-Lora A, et al. Teacher Job Stress and Satisfaction in Urban Schools: Disentangling Individual-, Classroom-, and Organizational-Level Influences. Behav Ther. 2018;49(4):494-508 DOI: 10.1016/j.beth.2017.11.011.

86. Pandey A, Mishra RM, Sahu D, Benara SK, Sengupta U, Paranjape RS, et al. Heading towards the Safer Highways: an assessment of the Avahan prevention programme among long distance truck drivers in India. BMC Public Health. 2011;11 Suppl 6(Suppl 6):S15 DOI: 10.1186/1471-2458-11-S6-S15.

87. Patterson PD, Moore CG, Guyette FX, Doman JM, Sequeira D, Werman HA, et al. Fatigue mitigation with SleepTrackTXT2 in air medical emergency care systems: study protocol for a randomized controlled trial. Trials. 2017;18 DOI: 10.1186/s13063-017-1999-z.

88. Patterson PD, Moore CG, Guyette FX, Doman JM, Weaver MD, Sequeira DJ, et al. Real-Time Fatigue Mitigation with Air-Medical Personnel: The SleepTrackTXT2 Randomized Trial. Prehosp Emerg Care. 2019;23(4):465-78 DOI: 10.1080/10903127.2018.1532476.

89. Patterson PD, Moore CG, Weaver MD, Buysse DJ, Suffoletto BP, Callaway CW, et al. Mobile phone text messaging intervention to improve alertness and reduce sleepiness and fatigue during shiftwork among emergency medicine clinicians: Study protocol for the SleepTrackTXT pilot randomized controlled trial. Trials. 2014;15(1) DOI: 10.1186/1745-6215-15-244.

90. Pesola AJ, Laukkanen A, Haakana P, Havu M, Saakslahti A, Sipila S, et al. Muscle Inactivity and Activity Patterns after Sedentary Time-Targeted Randomized Controlled Trial. Med Sci Sports Exerc. 2014;46(11):2122-31 DOI: 10.1249/mss.0000000000000335.

91. Pidd K, Roche A, Fischer J. A recipe for good mental health: A pilot randomised controlled trial of a psychological wellbeing and substance use intervention targeting young chefs. Drugs (Abingdon Engl). 2015;22(4):352-61 DOI: 10.3109/09687637.2015.1016400.

92. Portoghese I, Siddi M, Chessa L, Costanzo G, Garcia-Larsen V, Perra A, et al. COVID-19 Vaccine Hesitancy among Italian Healthcare Workers: Latent Profiles and Their Relationships to Predictors and Outcome. Vaccines (Basel). 2023;11(2):273 DOI: 10.3390/vaccines11020273.

93. Portugal LCL, Gama CMF, Gonçalves RM, Mendlowicz MV, Erthal FS, Mocaiber I, et al. Vulnerability and Protective Factors for PTSD and Depression Symptoms Among Healthcare Workers During COVID-19: A Machine Learning Approach. Front Psychiatry. 2021;12:752870 DOI: 10.3389/fpsyt.2021.752870.

94. Ratzon NZ, Bar-Niv NA, Froom P. The effect of a structured personalized ergonomic intervention program for hospital nurses with reported musculoskeletal pain: An assigned randomized control trial. Work. 2016;54(2):367-77 DOI: 10.3233/WOR-162340.

95. Robles-Granda P, Lin S, Wu X, Martinez GJ, Mattingly SM, Moskal E, et al. Jointly Predicting Job Performance, Personality, Cognitive Ability, Affect, and Well-Being. IEEE Comput Intell Mag. 2021;16(2):46-61 DOI: 10.1109/mci.2021.3061877.

96. Rollo S, Prapavessis H. A combined health action process approach and mHealth intervention to reduce workplace sitting time in office-working adults: a secondary analysis examining health-related quality of life and work performance outcomes. Psychol Health. 2021;36(10):1200-16 DOI: 10.1080/08870446.2020.1838522.

97. Sanders MR, Stallman HM, McHale M. Workplace Triple P: A controlled evaluation of a parenting intervention for working parents. J Fam Psychol. 2011;25(4):581-90 DOI: 10.1037/a0024148.

98. Schelleman-Offermans K, Ruiter RAC, Massar K. The Future Positive micro-intervention protocol: A program aiming to increase a healthy life-style among employees with a low socio-economic position. Front Public Health. 2022;10:832447 DOI: 10.3389/fpubh.2022.832447.

99. Seaton CL, Bottorff JL, Oliffe JL, Jones-Bricker M, Caperchione CM, Johnson ST, et al. Acceptability of the POWERPLAY Program: A Workplace Health Promotion Intervention for Men. Am J Mens Health. 2017;11(6):1809-22 DOI: 10.1177/1557988317728354.

100. Soliman GA, Kim J, Lee JM, High R, Hortman S, Kim Y, et al. Wellness programme at the workplace promotes dietary change and improves health indicators in a longitudinal retrospective study. Public Health Nutr. 2019;22(2):354-62 DOI: 10.1017/S1368980018002380. Epub 2018 Oct 1.

101. Somers M, Birnbaum D, Casal J. Application of the person-centered model to stress and well-being research: An investigation of profiles of employee well-being. Employee Relations. 2019;41(4):649-61 DOI: 10.1108/ER-06-2018-0154.

102. Somerset S, Evans C, Blake H. Accessing Voluntary HIV Testing in the Construction Industry: A Qualitative Analysis of Employee Interviews from the Test@Work Study. Int J Environ Res Public Health. 2021;18(8):4184 DOI: 10.3390/ijerph18084184.

103. Stephens SK, Eakin EG, Clark BK, Winkler EAH, Owen N, LaMontagne AD, et al. What strategies do desk-based workers choose to reduce sitting time and how well do they work? Findings from a cluster randomised controlled trial. Int J Behav Nutr Phys Act. 2018;15 DOI: 10.1186/s12966-018-0731-z.

104. Stephenson A, Garcia-Constantino M, Murphy MH, McDonough SM, Nugent CD, Mair JL. The “Worktivity” mHealth intervention to reduce sedentary behaviour in the workplace: a feasibility cluster randomised controlled pilot study. BMC Public Health. 2021;21(1):1416 DOI: 10.1186/s12889-021-11473-6.

105. Sun Y, Wang A, Yu S, Hagger MS, Chen X, Fong SSM, et al. A blended intervention to promote physical activity, health and work productivity among office employees using intervention mapping: a study protocol for a cluster-randomized controlled trial. BMC Public Health. 2020;20(1):994 DOI: 10.1186/s12889-020-09128-z.

106. Tan AM, LaMontagne AD, English DR, Howard P. Efficacy of a workplace osteoporosis prevention intervention: a cluster randomized trial. BMC Public Health. 2016;16(1):859 DOI: 10.1186/s12889-016-3506-y.

107. Tan AM, LaMontagne AD, Sarmugam R, Howard P. A cluster-randomised, controlled trial to assess the impact of a workplace osteoporosis prevention intervention on the dietary and physical activity behaviours of working women: study protocol. BMC Public Health. 2013;13:405 DOI: 10.1186/1471-2458-13-405.

108. Thogersen-Ntoumani C, Black J, Lindwall M, Whittaker A, Balanos GM. Presenteeism, stress resilience, and physical activity in older manual workers: a person-centred analysis. Eur J Ageing. 2017;14(4):385-96 DOI: 10.1007/s10433-017-0418-3.

109. Thorndike AN, McCurley JL, Gelsomin ED, Anderson E, Chang Y, Porneala B, et al. Automated Behavioral Workplace Intervention to Prevent Weight Gain and Improve Diet: The ChooseWell 365 Randomized Clinical Trial. JAMA Netw Open. 2021;4(6):e2112528 DOI: 10.1001/jamanetworkopen.2021.12528.

110. Tognetto A, Zorzoli E, Franco E, Gervasi G, Paglione L, Di Ninno F, et al. Seasonal influenza vaccination among health-care workers: the impact of different tailored programs in four University hospitals in Rome. Hum Vaccin and Immunother. 2020;16(1):81-5 DOI: 10.1080/21645515.2019.1632684.

111. Tshabalala AMET, Taylor M. An innovation to improve health outcomes in Amajuba district, KwaZulu-Natal, South Africa. Dev South Afr. 2018;35(4):497-510 DOI: 10.1080/0376835x.2018.1483225.

112. Tucker S, Farrington M, Lanningham-Foster LM, Clark MK, Dawson C, Quinn GJ, et al. Worksite Physical Activity Intervention for Ambulatory Clinic Nursing Staff. Workplace Health Saf. 2016;64(7):313-25 DOI: 10.1177/2165079916633225.

113. van Doorn D, Richardson N, Meredith D, Blake C, McNamara J. Study protocol: Evaluation of the 'real-world' Farmers Have Hearts - Cardiovascular Health Program. Prev Med Rep. 2022;30:102010 DOI: 10.1016/j.pmedr.2022.102010.

114. van Heijster H, van Berkel J, Bakker M, Boot C, de Vet E. Process evaluation of workplace health promotion in a sheltered workplace: a care ethics perspective. Health Promot Int. 2023;38(2):daad031 DOI: 10.1093/heapro/daad031.

115. van Holland BJ, de Boer MR, Brouwer S, Soer R, Reneman MF. Sustained employability of workers in a production environment: design of a stepped wedge trial to evaluate effectiveness and cost-benefit of the POSE program. BMC Public Health. 2012;12:1003 DOI: 10.1186/1471-2458-12-1003.

116. van Holland BJ, Reneman MF, Soer R, Brouwer S, de Boer MR. Effectiveness and Cost-benefit Evaluation of a Comprehensive Workers' Health Surveillance Program for Sustainable Employability of Meat Processing Workers. J Occup Rehabil. 2018;28(1):107-20 DOI: 10.1007/s10926-017-9699-9

117. Verburgh M, Verdonk P, Appelman Y, Brood-van Zanten M, Hulshof C, Nieuwenhuijsen K. Workplace Health Promotion Among Ethnically Diverse Women in Midlife With a Low Socioeconomic Position. Health Educ Behav. 2022;49(6):1042-55 DOI: 10.1177/10901981211071030.

118. Viester L, Verhagen E, Proper KI, van Dongen JM, Bongers PM, van der Beek AJ. VIP in construction: systematic development and evaluation of a multifaceted health programme aiming to improve physical activity levels and dietary patterns among construction workers. BMC Public Health. 2012;12 DOI: 10.1186/1471-2458-12-89.

119. Viester L, Verhagen EALM, Bongers PM, van der Beek AJ. Effectiveness of a worksite intervention for male construction workers on dietary and physical activity behaviors, body mass index, and health outcomes: Results of a randomized controlled trial. Am J Health Promot. 2018;32(3):795-805 DOI: 10.1177/0890117117694450.

120. von Lengerke T, Lutze B, Krauth C, Lange K, Stahmeyer JT, Chaberny IF. Promoting Hand Hygiene Compliance: PSYGIENE-a Cluster-Randomized Controlled Trial of Tailored Interventions. Dtsch Arztebl Int. 2017;114(3):29-36 DOI: 10.3238/arztebl.2017.0029.

121. Walker N, Dobbing R. A redesigned training and staff support programme to enhance job retention in employees with moderate-severe depression. Mental Health Soc Incl. 2021;25(3):279-95 DOI: 10.1108/mhsi-12-2020-0089.

122. Wallis A, Robertson J, Bloore RA, Jose PE. Differences and siminlarities between leaders and nonleaders on psychological distress, wellbeing, and challenges at work. Consult Psychol J. 2021;73(4):325-48 DOI: 10.1037/cpb0000214.

123. Walthouwer MJL, Oenema A, Soetens K, Lechner L, De Vries H. Implementation of web-based interventions by Dutch occupational health centers. Health Promot Int. 2017;32(5):818-30 DOI: 10.1093/heapro/daw014.

124. Watkins C, Macy G, Golla V, Lartey G, Basham J. The Total Worker Health Concept: A Case Study in a Rural Workplace. J Occup Environ Med. 2018;60(5):387-91 DOI: 10.1097/JOM.0000000000001273.

125. Wollesen B, Hagemann D, Pabst K, Schlüter R, Bischoff LL, Otto AK, et al. Identifying individual stressors in geriatric nursing staff-a cross-sectional study. International Journal of Environmental Research and Public Health. 2019;16(19) DOI: 10.3390/ijerph16193587.

126. Zhai D, Schiavone G, Van Diest I, Vrieze E, DeRaedt W, Van Hoof C. Ambulatory Smoking Habits Investigation based on Physiology and Context (ASSIST) using wearable sensors and mobile phones: Protocol for an observational study. BMJ Open. 2019;9(9):e028284 DOI: 10.1136/bmjopen-2018-028284.

127. Zhang E, Dai Z, Wang C, Hu J, Wang S, Zhang L, et al. Targeting COVID-19 vaccine hesitancy among nurses in Shanghai: A latent profile analysis. Front Public Health. 2022;10:953850 DOI: 10.3389/fpubh.2022.953850.

128. Zhang Y, Cordina-Duverger E, Komarzynski S, Attari AM, Huang Q, et al. Digital circadian and sleep health in individual hospital shift workers: A cross sectional telemonitoring study. EBioMedicine. 2022;81:104121 DOI: 10.1016/j.ebiom.2022.104121.

129. Zoellner J, You W, Almeida F, Blackman KCA, Harden S, Glasgow RE, et al. The Influence of Health Literacy on Reach, Retention, and Success in a Worksite Weight Loss Program. Am J Health Promot. 2016;30(4):279-82 DOI: 10.1177/0890117116639558.
